# Primary Adrenal Gland Lymphoma: Report of 13 Cases—A Retrospective Multicenter Polish Lymphoma Research Group Analysis

**DOI:** 10.3390/life16020230

**Published:** 2026-02-01

**Authors:** Magdalena Witkowska, Kacper Kościelny, Agnieszka Giza, Ryszard Swoboda, Joanna Drozd-Sokołowska, Dariusz Wołowiec

**Affiliations:** 1 Department of Hematology, Medical University of Lodz, 93-510 Lodz, Poland; magdamalicka@gmail.com (M.W.); kacper.koscielny@umed.lodz.pl (K.K.); 2 Department of Biostatistics and Translational Medicine, Medical University of Lodz, 92-215 Lodz, Poland; 3 Department of Haematology, Uniwersytet Jagiellonski w Krakowie Collegium Medicum, 30-688 Krakow, Poland; agnieszka.giza4@wp.pl; 4 Bone Marrow Transplant and Oncohematology Clinic, 44-102 Gliwice, Poland; ryszard.swoboda@gliwice.pl; 5 Department of Hematology, Oncology and Internal Diseases, Medical University of Warsaw, 02-097 Warsaw, Poland; johna.dr@poczta.fm; 6 Department of Hematology, Cellular Therapies and Internal Diseases, Wrocław Medical University, 50-567 Wrocław, Poland

**Keywords:** primary adrenal gland lymphoma, non-Hodgkin lymphoma, extranodal lymphomas

## Abstract

**Introduction:** The existence of primary adrenal gland lymphoma (PAGL) has been debated due to lack of lymphoid tissue in the adrenal glands. PAGL is extremely rare, accounting for less than 1% of all types of lymphomas. The aim of this study was to analyze patients with PAGL in Polish population. **Material and Methods:** We retrospectively reviewed 13 adult patients with PAGL diagnosed in Polish Hematological Centers. **Results:** A total of 13 patients (5 women and 8 men) with PAGL were included into the study. The median age at the diagnosis was 69.1 years (range: 31–85). The most common histological type was diffuse large B-cell lymphoma (DLBCL)-12 patients, the remaining one was diagnosed with Hodgkin lymphoma (HL). In 7 patients (54%), the left adrenal gland was involved; in 3 patients (23.5%), the right adrenal gland was involved; and 3 patients (23.5%) had bilateral lymphoma. Systemic symptoms (B symptoms) were observed in 11 out of 13 patients (85%). Two patients (15%) were treated with chemotherapy alone and the remaining eleven patients (85%) with immune and chemotherapy together (85%). During the follow-up period, 11 patients died, 8 had relapsed or refractory disease (62%), and 3 patients (23%) had relapse in central nervous system (CNS). The median progression-free survival (PFS) was 14.63 months, while the median overall survival (OS) was 20.30 months. Adrenalectomy of the involved adrenal gland was associated with shorter PFS (*p* = 0.0165), with trend of shorter OS. Achieving complete remission (CR) after front line treatment was associated with significantly longer OS (*p* = 0.0239) and PFS (*p* = 0.0152). **Conclusions:** Adrenal glands are extremely rare as primary locations of extranodal lymphoma. The prognosis of PAGL is generally poor. In this study, we described demographic, clinical, and pathological characteristics as well as factors that may affect survival among these groups. So far, it is the largest polish multicenter experience describing patients with PAGL.

## 1. Introduction

The incidence of secondary adrenal involvement in non-Hodgkin lymphoma (NHL) is approximately 5% but that incidence rises up to 35% when autopsy cases are included [1]. Primary adrenal gland lymphoma (PAGL) is a particularly rare location of primary extranodal lymphoma, accounting for 1% of all NHL cases [2]. PAGL occurs predominantly in males, with a male-to-female ratio of 7:1 [3]. The average age at the diagnosis is 70 years [4]. The etiology of PAGL is still not clear. The most common hypothesis involves hematopoietic tissue akin to adrenal myelolipoma resting in the adrenal glands [5]. It is believed that the adrenal glands contain no lymphoid tissue and that lymphoma may arise on a background of previous autoimmune adrenalitis. It is consistent with the findings of primary adrenal insufficiency being usually observed in bilateral PAGL patients [6,7].

Due to the extreme rarity of this type of lymphoma, differential diagnosis may be difficult [8]. PAGL may be clinically silent and detected as incidentalomas. Among PAGL patients, most cases are diffuse large B-cell lymphoma (DLBCL) (more than 70%), majority of a non-germinal center B-cell (non-GCB) phenotype. Currently, there is no strict definition of PAGL. It is widely accepted among experts that diagnosis requires lymphomatous involvement of one or both adrenal glands, with no prior history of lymphoma. In patients with extra-adrenal involvement, the adrenal glands must be the unequivocal dominant lesion [9]. The most common symptoms are systemic symptoms (B symptoms) [4]. Other symptoms such as lumbar pain, adrenal insufficiency, and fatigue are reported with lower frequencies.

Given the rarity of the disease, no prospective studies aiming for the optimal management of PAGL have been reported. So far, only few series reported dismal outcomes, with a 12-month survival rate of around 20%. Unresolved therapeutic issues include the role of bilateral adrenalectomy and/or adjuvant radiation therapy. Moreover, there are no standards regarding central nervous system (CNS) prophylaxis. Given recent reports, there is a high risk of CNS relapse in PAGL patients. According to a recent multicenter case series of 50 patients, outcome remains poor with 2-year overall survival (OS) at 61.5% [10].

So far, about 250 cases of PAGL are reported in the world literature [11]. Due to the small number of relevant studies, data concerning this type of extranodal NHL are very limited. The aim of this study was to analyze all PAGL patients in Polish Lymphoma Research Group (PLRG) centers between 2005 and 2025, particularly demographic data, histological type, risk factors, front line treatment, and their possible impact on survival parameters.

## 2. Patients and Methods

We retrospectively analyzed 13 patients diagnosed with PAGL, treated in five Polish hematology/oncology centers between January 2005 and June 2025. Clinical and laboratory data were extracted from institutional medical records.

**Figure 1:**
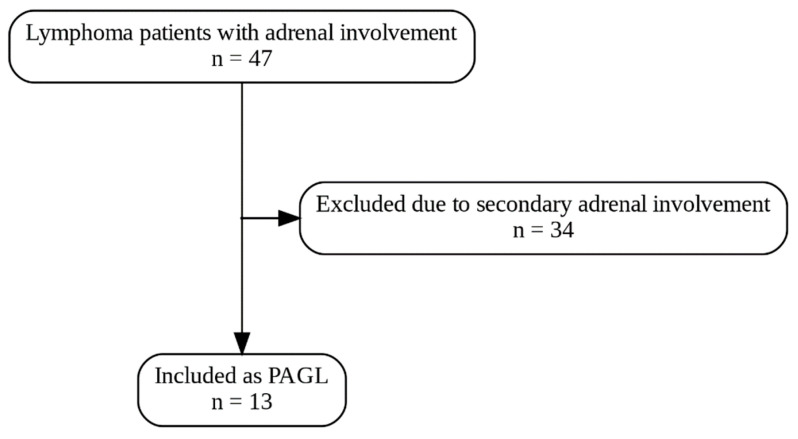
Flowchart of patients screened for primary adrenal gland lymphoma.

Histological diagnosis was established according to the World Health Organization (WHO) lymphoma classification used in the year of diagnosis. DLBCL was further classified into germinal center B-cell (GCB) and non-GCB subtypes by the Hans algorithm bases on the positivity of three markers (CD10, MUM1/IRF4, and BCL6). The GCB subtype was characterized by CD10^+^/MUM1^−^, CD10^+^/MUM1^+^, or CD10^−^/MUM1^−^/BCL6^+^. The non-GCB subtype was characterized by CD10^−^/BCL6^−^ and CD10^−^/MUM1^+^/BCL6^+^ cases. The diagnostic criteria included the following: a histologically proven lymphoma, no prior history of lymphoma, with or without only regional lymph node involvement, and dominant adrenal lesions in the presence of other organs and/or lymph nodes metastases. Patients with generalized lymphadenopathy or secondary adrenal infiltration were excluded from the study (Figure 1).

Disease staging was established according to the Ann Arbor classification. The CNS International Prognostic Index (CNS-IPI) was evaluated in all DLBCL patients. Treatment responses were assessed based on the Lugano 2014 criteria: complete remission (CR) was defined as complete resolution of all target lesions on imaging with normalization of FDG-PET uptake (Deauville score 1–3), while partial remission (PR) was defined as a ≥50% reduction in the sum of the product of diameters (SPD) of target lesions, without the appearance of new lesions or progression of existing ones.

Data on presenting symptoms, imaging findings, laboratory abnormalities, treatment modalities, and outcomes were collected and analyzed. All histopatological diagnoses were confirmed using standard diagnostic procedures.

The study was conducted in accordance with institutional ethics standards. All data were fully anonymized prior to analysis. Given that the study was observational and retrospective according to the Polish legislation the approval of Ethic Committee or individual informed consents were not required.

## 3. Statistical Analysis

Nominal variables were summarized as counts and percentages, based on the number of patients with available data. Continuous variables were presented as medians with interquartile ranges (IQRs), depending on the distribution of the data. Median follow-up was estimated using the reverse Kaplan–Meier method. Progression-free survival (PFS) and OS were calculated using the Kaplan–Meier method, and corresponding 95% confidence intervals were reported. Survival analyses were performed in 12 patients, as one patient was lost to follow-up. Survival comparisons between groups were initially assessed using the log-rank test. For clarity and readability, only comparisons that reached statistical significance are presented in the manuscript, given the small sample size and the exploratory nature of the study. However, all group comparisons were performed, and non-significant results are available upon request. All statistical analyses were conducted using Statistica Version 13.1 (TIBCO Software Inc., Palo Alto, CA, USA) and Python programming language (version 3.12.12).

## 4. Results

### Patient Characteristics

Thirteen patients (eight males, five females) with diagnosis of PAGL were included into the study. The median age at diagnosis was 69.1 years (range: 31–85). The histopathological diagnosis was based on histopathological examination and immunohistochemistry of the adrenal tissue following adrenal biopsy or specimens from adrenalectomy. In 12 (92.3%) patients, the diagnosis was DLBCL, and 9 of them (75%) were non-GCB subtype. One patient (7.7%) had classical Hodgkin lymphoma (HL) nodular sclerosis subtype.

At diagnosis, 11 patients (84.6%) exhibited systemic symptoms including fever (over 38 °C), night sweats, and significant unintentional weight loss (over 10% body weight in 6 months). Other most frequent symptoms at presentation were as follows: abdominal tumor (38.5%, n = 5), followed by abdominal pain (15.4%, n = 2) and fatigue (15.4%, n = 2). In one case (7.7%) the diagnosis was made incidentally as pathological mass was observed in routine ultrasonography (USG).

Diagnostic imaging varied across the cohort: CT was used in 3 patients (23.1%)—in 1 patient, PET/CT was performed, and in 2 cases, CT was made alone. PET-CT was used in 11 cases (84.6%) and additional USG in 2 patients (15.4%). The left adrenal gland was the most common site of tumor location (54%, n = 7). Right adrenal gland was infiltrated in 3 patients (23%), and bilateral adrenal involvement was observed in 3 cases (23%).

Regional lymph node involvement was detected in 69.2% of patients (n = 9). Distant lymph node involvement excluded patients from the study. Three patients (23.1%) had infiltration of surrounding adjacent tissues with the main mass in adrenal gland confirmed by PET/CT scans. Four patients had only adrenal involvement, with all non-GCB type. Among this group, 2 patients had CR (15.4%) and 2 exhibited no response (NR) (15.4%) after front line treatment. The median tumor diameter exceeded 5 cm in 46.2% (n = 6). Data on exact tumor size were not reported in 7 patients (54%).

Laboratory findings at diagnosis showed elevated serum lactate dehydrogenase (LDH) activity in 84.6% of cases (n = 11), hypoalbuminemia in 15.4% (n = 2), and serum elevated β-2 microglobulin concentration in 38.5% (n = 5). No cases of moderate or severe anemia (HGB < 10 g/dL), thrombocytopenia, or impaired renal function (creatinine > 1.5 mg/dL) were documented. Detailed data are shown in Table 1, Table 2 and Table 3.

**Table 1:** Cases profiles, chemotherapy regiments, CNS-IPI, prophylaxis, and survival outcome patients with primary adrenal gland lymphoma.

Nr	Sex	Age	Diagnosis	Symptoms	First-Line Regimen	CNS-IPI	CNS Prophylaxis	Response After 1st Line
1	M	70	GCB DLBCL	Incidentaloma	6 cycles of R-CHOP	Inter	3× Mtx i.t.	CR
2	M	56	Non-GCB DLBCL	Lumbosacral pain, fever, fatigue	8 cycles of CHOP	Inter	2× Mtx i.t.	NR
3	F	81	Non-GCB DLBCL	N/A	4 cycles of R-CHOP	Low	no	N/A
4	M	69	GCB DLBCL	Weight loss	6 cycles of R-CHOP	High	2 cycles of Mtx i.v.	CR
5	M	63	Non-GCB DLBCL	Bone pain, weight loss	6 cycles of R-CHOP	High	Mtx i.v.	CR
6	M	61	GCB DLBCL	Weight loss	8 cycles of R-CHOP	Low	no	CR
7	M	71	Non-GCB DLBCL	Proximal myopathy, fever	5 cycles of R-CHOP	Inter	4× Mtx i.t.	PD
8	M	79	Non-GCB DLBCL	Abdominal tumor, night sweats	8 cycles of miniR-CHOP	Low	no	PR
9	F	50	Non- GCB DLBCL	Vertigo, abdominal tumor, fever	5 cycles of DA-EPOCH-R	Inter	2 cycles of Mtx i.v.	PR
10	F	80	Non-GCB DLBCL	Fever, abdominal tumor	2 cycles of miniR-CHOP + radiotherapy	Inter	no	NR
11	F	85	Non-GCB DLBCL	Abdominal pain, night sweats, abdominal tumor	5 cycles of CHOP	Inter	no	NR
12	F	31	Non-GCB DLBCL	Abdominal pain, abdominal tumor, weight loss	6 cycles of R-CHOP + ASCT	Inter	2 cycles of Mtx i.v.	CR
13	M	41	HL	Night sweats, fatigue	6 cycles of A-AVD	-		CR

Abbreviations: **M**, male; **F**, female; **GCB**, germinal center B-cell-like; **DLBCL**, diffuse large B-cell lymphoma; **HL**, Hodgkin lymphoma; **CNS-IPI**, Central Nervous System International Prognostic Index (score 0–1: low risk; score 2–3: intermediate risk; score 4–6: high risk); **CNS**, central nervous system; **i.t.**, intrathecal; **i.v.**, intravenous; **Mtx**, methotrexate; **R-CHOP**, rituximab, cyclophosphamide, doxorubicin, vincristine, and prednisone; **CHOP**, cyclophosphamide, doxorubicin, vincristine, and prednisone; **miniR-CHOP**, dose-reduced R-CHOP; **DA-EPOCH-R**, dose-adjusted etoposide, prednisone, vincristine, cyclophosphamide, doxorubicin, and rituximab; **A-AVD**, brentuximab vedotin, doxorubicin, vinblastine, and dacarbazine; **ASCT**, autologous stem cell transplantation; **CR**, complete remission; **PR**, partial remission; **NR**, no response; **PD**, progressive disease; **N/A**, not available.

**Table 2:** Demographic and clinical characteristics of patients with PAGL.

Variable		N (%)
Number		13 (100%)
Age at diagnosis	69.1	(range: 31–85)
Sex	F: 5 (38.5%)	M: 8 (61.5%)
Symptoms (excluding “B” symptoms)	Abdominal tumor	5 (38.5%)
	Abdominal pain	2 (15.4%)
	Fatigue	2 (15.4%)
	Lumbosacral pain	1 (7.7%)
	Vertigo	1 (7.7%)
	Proximal myopathy	1 (7.7%)
	Bone pain	1 (7.7%)
Incidental discovery		1 (7.7%)
Systemic symptoms		11 (84.6%)
Localization of the tumor	Left adrenal only	7 (54%)
	Right adrenal only	3 (23%)
	Bilateral	3 (23%)
Lymph node involvement	Only regional	9 (69.2%)
Type of lymphoma	DLBCL	12 (92.3%)
	HL	1 (7.7%)
First-line treatment	R-CHOP	7 (54%)
	miniRCHOP	2 (15.3%)
	DA-EPOCH-R	1 (7.7%)
	CHOP	2 (15.3%)
	A-AVD	1 (7.7%)
Radiotherapy		1 (7.7%)
ASCT		1 (7.7%)
Response to the first-line treatment	CR	6 (50%)
	PR	2 (16.7%)
	PD/NR	4 (33.3%)

Data are presented as n (%), indicating the number of patients and the percentage of the total study cohort. Age at diagnosis is reported as mean with age range. B symptoms include fever, night sweats, and unintentional weight loss. DLBCL, diffuse large B-cell lymphoma; HL, Hodgkin lymphoma; R-CHOP, rituximab, cyclophosphamide, doxorubicin, vincristine, and prednisone; miniR-CHOP, dose-reduced R-CHOP; DA-EPOCH-R, dose-adjusted etoposide, prednisone, vincristine, cyclophosphamide, doxorubicin, and rituximab; CHOP, cyclophosphamide, doxorubicin, vincristine, and prednisone; A-AVD, brentuximab vedotin, doxorubicin, vinblastine, and dacarbazine; ASCT, autologous stem cell transplantation; CR, complete remission; PR, partial remission; PD, progressive disease; NR, no response.

**Table 3:** Summary table of survival outcomes.

Group	N (%)	Median OS (Months)	Median PFS (Months)
Overall	12 (100%)	20.3	14.6
Surgery	5 (41.7%)	8.3	6.5
Without surgery	7 (58.3%)	20.3	15.9
CR	6 (50.0%)	38.2	15.9
No CR	6 (50.0%)	6.5	4.2

Among 13 PAGL patients, the most commonly used front line treatment was immune and chemotherapy including rituximab, cyclophosphamide, doxorubicin, vincristine, and prednisone (R-CHOP) in 7 patients (54%), reduced dose rituximab, cyclophosphamide, doxorubicin, vincristine, and prednisone (miniRCHOP) in 2 patients (15.3%), chemotherapy alone including cyclophosphamide, doxorubicin, vincristine, and prednisone (CHOP) in 2 patients (15.3%). One patient was treated with dose-adjusted etoposide, prednisone, vincristine, cyclophosphamide, doxorubicin, and rituximab (DA-EPOCHR). One patient with HL diagnosis was treated with brentuximab vedotin with doxorubicin, vinblastine, and dacarbazine (A-AVD). Adrenalectomy of involved gland was performed in 5 cases (38.5%)—4 with left adrenal gland and 1 with right adrenal gland. One patient treated with DA-EPOCHR underwent autologous hematopoietic stem cell transplantation (autoHSCT) in CR as consolidative treatment and one had radiotherapy on involved fields after 2 cycles of miniRCHOP due to severe adverse events during treatment.

CNS-IPI was calculated in all patients with DLBCL diagnosis. CNS-IPI was low in 2 (15.3%) patients, intermediate in 7 (54%) patients, and high in 2 (15.3%) patients. Patients with low CNS-IPI did not receive CNS prophylaxis. Among 7 patients with intermediate CNS-IPI, 3 patients were treated with intathecal (i.t.) methotrexate (Mtx) and 2 with intravenous (i.v.) high-dose (HD) Mtx. Two patients with high CNS-IPI were also treated with HD-Mtx i.v.

Treatment responses were evaluable in 12 patients. CR was achieved in 6 (50%), PR in 2 (16.7%), and primary resistance or progressive disease (PD) and NR was noted in 4 (33.3%). Progression in CNS was observed in three patients (23%)—one patient had high CNS-IPI and was treated with HD-Mtx. Two other patients had intermediate CNS-IPI—one was treated with i.t. Mtx and one did not receive any prophylaxis.

## 5. Survival Analysis

Median follow-up period, calculated using the reverse Kaplan–Meier method, was 24.8 months (Q1: 12.7 months, Q3: not reached).

Median PFS was 14.63 months (95% CI: 3.13–15.90), and the median OS was 20.30 months (95% CI: 3.13–20.47) (Figure 1). At the end of follow-up, no patients were alive. In all patients, the death was related to lymphoma. One patient was lost to follow-up.

**Figure 2:**
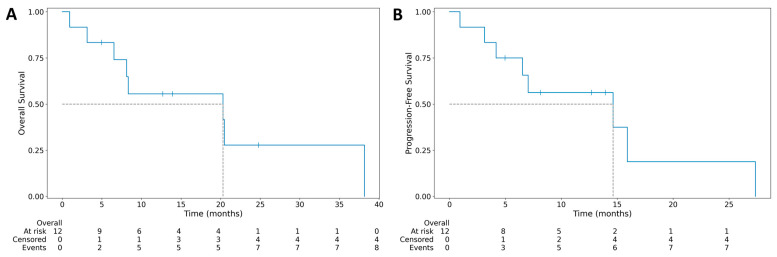
(**A**) Median overall survival for 12 cases of primary adrenal gland lymphoma. (**B**) Median progression-free survival for 12 cases of primary adrenal gland lymphoma.

Adrenalectomy of involved gland was associated with shorter PFS (log-rank *p* = 0.0165) and with a no significant trend toward inferior OS (Figure 2).

**Figure 3:**
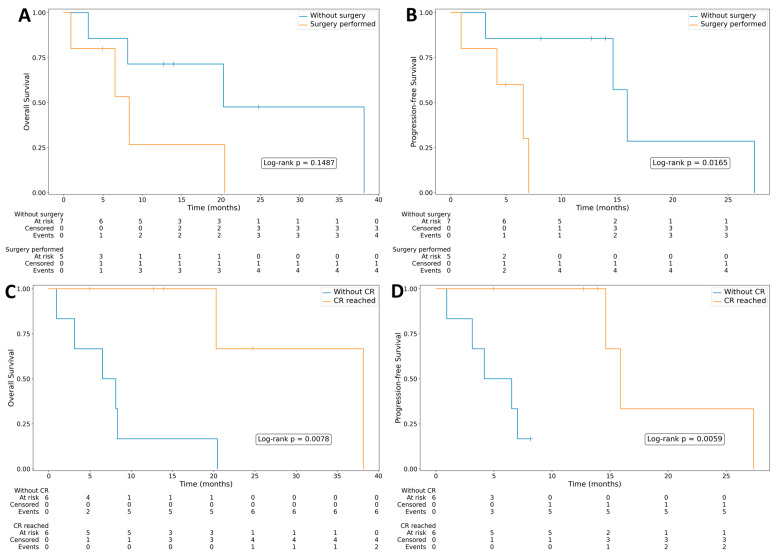
(**A**) Overall survival in groups with or without surgery performed-(total adrenalectomy of involved gland). (**B**) Progression-free survival in groups with or without surgery performed. (**C**) Overall survival in groups with or without complete remission. (**D**) Progression-free survival in groups with or without complete remission.

Achieving CR was significantly associated with longer both PFS (log-rank *p* = 0.0152) and OS (log-rank *p* = 0.0239) (Figure 3).

## 6. Discussion

PAGL is an extremely rare type of extranodal lymphoma, with around 250 cases reported so far. The literature regarding PAGL consists mostly of case reports and small series. Among PAGL, most cases are DLBCL, with only rare cases of plasmablastic lymphoma, extranodal NK/T-cell nasal type, and HL described [12]. According to the data published so far, most primary adrenal DLBCL are non-GCB phenotypes. Our data show a similar observation, since 12 out of 13 patients had DLBCL, and 75% of them were a non-GCB subtype. This subtype is biologically distinct and is known to have less favorable prognosis. The non-GCB subtype is often associated with a more aggressive course of disease with lower survival rates. It may, at least partially, explain unsatisfactory treatment results in PAGL cohort. PAGL is usually observed in elderly, predominantly male patients, with a median age at presentation ranging from 48 to 68 years and a male/female ratio of 1.8:1–7:1 [13]. Our results are similar as majority of patients were male (61.5%), with the median age at diagnosis being 69.1 years (range: 31–85). Dobrinja et al. suggest that the highest incidence occurs in adults approximately 60 years old, which corresponds to the presented series [14].

To the best of our knowledge, so far there are six cases of primary adrenal HL to be reported in the literature [15]. In the published data, no patients experienced B symptoms or adrenal insufficiency [16]. These findings are different than data presented for DLBCL PAGL patients. Our patient had fever at the time of diagnosis. Furthermore, the median reported age is around 60, as our patient was much younger (41 years old). Based on these limited cases, unilateral distribution is more frequent and equal sex distribution was observed. HL is an extremely rare PAGL manifestation, which is difficult to diagnose and treat. Moreover, the disease is so unique that no further conclusions can be made.

Simultaneous involvement of both adrenal glands is very common, and accounts for 75% of previously reported cases [17]. In contrast, in our study, bilateral adrenal involvement was observed only in 3 patients (23%). The most common was left adrenal gland involvement in 7 cases (54%). It is possible that these differences may be due to small sample size, which was main limitation of this study.

Patients with bilateral adrenal gland involvement usually develop adrenocortical insufficiency. In a retrospective multicenter study by Majidi et al. [18], almost half of PAGL patients have bilateral adrenal gland involvement with coexistence of adrenal insufficiency. In a study by Laurent et al. [19], approximately 43% of patients with bilateral PAGL have symptoms of adrenocortical insufficiency, such as hypotension, vomiting, and hyponatremia. In patients with unilateral involvement, adrenocortical insufficiency is not usually observed. The possible cause of a lack of excretory endocrine function in bilateral PAGL is diffuse lymphomatous infiltration, present in more than 90% of adrenal tissues. It leads to structural destruction of the adrenal gland caused by lymphoma cells [20]. There are few reports on the incidence of adrenal insufficiency in adrenal metastases of solid tumors, which makes comparative analyses of PAGL highly problematic [21,22]. In our series, no laboratory tests of adrenal gland hormonal activity were performed, as there were no clinical symptoms of adrenal insufficiency. Lack of information about adrenocortical insufficiency is a major limitation of this study. Consequently, endocrinology evaluation should be performed in each patient with PAGL. Moreover, systematic endocrinological assessment should also be considered in all suspected PAGL cases, regardless of overt clinical symptoms of adrenal insufficiency.

In the literature, immune system dysfunction is proposed as a possible etiology of PAGL. Wang et al. reviewed 55 patients with PAGL and found that 13% had concomitant autoimmune disease [4]. None of our patients had any prior history of adrenal carcinoma or autoimmune disease, which is in accordance with data published by Rashidi et al. [13]. The systemic symptoms called “B” symptoms (unexplained fever, unintentional weight loss >10% in 6 months, night sweats) are reported in the majority of patients with PAGL (68%) [23]. Our data show similar observation, since B symptoms occurred in 85% of patients. In contrast, in our series, abdominal pain and fatigue were less frequent (15%) than previously reported by Rashidi et al. (42% and 36%, respectively) [13].

There is no consensus regarding the specific management of PAGL, and protocols used for NHL of corresponding histological type are usually used. For the previously reported cases, different strategies, including adrenalectomy as well as immune and chemotherapy, are reported. In one of the largest series of PAGL, 31 patients received R-CHOP chemotherapy, achieving CR and overall response rate (ORR) of 54.8% and 87.0%, respectively [24]. In our study, the majority of DLBCL patients were treated with R-CHOP-like therapy. Our results were worse compared to data published so far, since ORR was 67.7%, and CR was achieved in 50% of patients.

R-CHOP-like regimen is still a standard first-line treatment for extranodal DLBCL patients including PAGL. The prognosis for this group is extremely poor, and its 5- and 10-year OS rates were reported to be as low as 19.17% and 3.33%, respectively [5]. In our study, the median PFS was 14.63 months, and the median OS was 20.30 months. In previous publications, combinations of surgery with chemotherapy or other treatments were reported. However, we observed that previous adrenalectomy shortened the PFS and was associated with a trend toward inferior OS. The possible reason may be adrenalectomy performed in patients with larger, symptomatic, or diagnostically uncertain lesions. As the number of patients is small, these results should be treated carefully. Therefore, the decision of adrenal gland resection before (immune)chemotherapy must be considered with great caution.

Disappointing results of the PAGL treatment with immunochemotherapy raises the question of the utility of the consolidation by megachemotherapy supported by autoHSCT or radiotherapy. In a study including 28 patients with PAGL, 64% were treated with a CHOP-based regimen, and 50% with R-CHOP protocol. They received autoHSCT, which allowed 1-year OS ratio 61.6% [6,7]. This result may be considered as a valid option for patients with this specific extranodal NHL.

The exact role of radiation therapy in the treatment of PAGL is unclear. Prolonged remission is observed in some patients with low-grade lymphoma as adrenalectomy was not complete [25]. In our study, one patient had radiation as a consolidation treatment after 2 cycles of miniRCHOP but had progressive disease. For sure, more data from clinical trials are required to assess the role of radiotherapy in PAGL patients.

It is believed that in PAGL patients, risk of CNS relapse is high. In patients with high and intermediate CNS-IPI score, CNS recurrence may be reduced with CNS prophylaxis. Currently available prophylaxis strategies, including i.t. Mtx and i.v. HD-Mtx, have yielded mixed results [26]. However, the findings are not yet verified in PAGL patients. In our study, CNS relapse was high and was observed in 3 patients (23%). One was treated with HD-Mtx i.v. but had a high CNS-IPI score, one had i.t. Mtx, and one did not receive prophylaxis. As the CNS relapse among PAGL patients is high, decision-making regarding prophylaxis should be considered in intermediate- and high-risk patients.

The major limitation of this study includes its retrospective character. Small sample size and incomplete laboratory test concerning adrenal insufficiency further limit the conclusions that can be drawn. Particularly in the context of bilateral disease and enlarged lymph nodes, diffuse involvement should be considered. Our study excluded these patients to maintain methodological consistency by focusing only on PAGL. Furthermore, molecular profiling is lacking in retrospective data. Further studies with larger, multicenter prospective studies should be addressed regarding this important diagnostic group.

## 7. Conclusions

In conclusion, PAGL is an extremely rare type of extranodal lymphoma. Additional research is needed to enhance the management of this lesion and attract the attention of clinicians. PAGL with poor prognosis must be diagnosed at an early stage to improve survival [27]. Standard first-line treatment dedicated to aggressive lymphoma, like R-CHOP-like protocols, are not effective enough. It seems that more aggressive protocols should be considered in fit patients including autoHSCT [28]. Although our series is limited by its small sample size, the observed trend towards prolonged survival in patients achieving CR further strengthens the value of effective first-line treatment.

## Data Availability

The original contributions presented in this study are included in the article. Further inquiries can be directed to the corresponding author.
